# Association Analysis of Deep Genomic Features Extracted by Denoising Autoencoders in Breast Cancer

**DOI:** 10.3390/cancers11040494

**Published:** 2019-04-07

**Authors:** Qian Liu, Pingzhao Hu

**Affiliations:** 1 Department of Biochemistry and Medical Genetics, College of Medicine, Faculty of Health Sciences, University of Manitoba, Winnipeg, MB R3E 0J9, Canada; qianl@myumanitoba.ca; 2 Research Institute in Oncology and Hematology, CancerCare Manitoba, Winnipeg, MB R3E 0V9, Canada; 3 Department of Computer Science, University of Manitoba, Winnipeg, MB R3T 2N2, Canada

**Keywords:** denoising autoencoders, breast cancer, feature extraction and interpretation, concatenated deep feature

## Abstract

Artificial intelligence-based unsupervised deep learning (DL) is widely used to mine multimodal big data. However, there are few applications of this technology to cancer genomics. We aim to develop DL models to extract deep features from the breast cancer gene expression data and copy number alteration (CNA) data separately and jointly. We hypothesize that the deep features are associated with patients’ clinical characteristics and outcomes. Two unsupervised denoising autoencoders (DAs) were developed to extract deep features from TCGA (The Cancer Genome Atlas) breast cancer gene expression and CNA data separately and jointly. A heat map was used to view and cluster patients into subgroups based on these DL features. Fisher’s exact test and Pearson’ Chi-square test were applied to test the associations of patients’ groups and clinical information. Survival differences between the groups were evaluated by Kaplan–Meier (KM) curves. Associations between each of the features and patient’s overall survival were assessed using Cox’s proportional hazards (COX-PH) model and a risk score for each feature set from the different omics data sets was generated from the survival regression coefficients. The risk scores for each feature set were binarized into high- and low-risk patient groups to evaluate survival differences using KM curves. Furthermore, the risk scores were traced back to their gene level DAs weights so that the three gene lists for each of the genomic data points were generated to perform gene set enrichment analysis. Patients were clustered into two groups based on concatenated features from the gene expression and CNA data and these two groups showed different overall survival rates (*p*-value = 0.049) and different ER (Estrogen receptor) statuses (*p*-value = 0.002, OR (odds ratio) = 0.626). All the risk scores from the gene expression and CNA data and their concatenated one were significantly associated with breast cancer survival. The patients with the high-risk group were significantly associated with patients’ worse outcomes (*p*-values ≤ 0.0023). The concatenated risk score was enriched by the AMP-activated protein kinase (AMPK) signaling pathway, the regulation of DNA-templated transcription, the regulation of nucleic acid-templated transcription, the regulation of apoptotic process, the positive regulation of gene expression, the positive regulation of cell proliferation, heart morphogenesis, the regulation of cellular macromolecule biosynthetic process, with FDR (false discovery rate) less than 0.05. We confirmed DAs can effectively extract meaningful genomic features from genomic data and concatenating multiple data sources can improve the significance of the features associated with breast cancer patients’ clinical characteristics and outcomes.

## 1. Introduction

Advanced hardware technologies have highly increased computational power, which makes the implementation of computation-consuming algorithms possible. At the same time, the development of biological technologies has greatly reduced the cost of genomic sequencing, which produced a huge amount of high-dimensional genomic data. Under these circumstances, bioinformatics becomes an exciting research field for researchers to explore the possibility to interpret genomic data using advanced computational technologies [1].

Different types of high-dimensional genomic data have been associated with cancer clinical characteristics and outcomes. The most commonly used ones are gene expression data and copy number alteration (CNA) data [2]. The activity of gene expression in tumor tissues is quite different from that in normal tissues [3] and has been established to have the ability to distinguish the characteristics of cancers [4]. There are some repeated segments in normal DNA, and during the process of cancer development, the repeated number of the segments may be changed due to abnormal DNA replication in tumor cells. This phenomenon is called copy number alteration [5]. CNA may result in chromosome structure changes in the forms of duplication or deletion in DNA segments. It has been shown that CNA plays an important role in the development of many types of cancers including breast cancer [6]. Therefore, it is highly necessary to mine the prognostic and diagnostic significance of the genome-wide cancer genomic data. From a clinical point of view, the prognosis of the genomic factors is always a necessary consideration because of its importance in making treatment plans [7]. In previous studies, prognosis significance was evaluated mainly based on clinical features, such as tumor grades and tumor subtypes [7] and molecular features, such as expression related gene signatures (e.g., PAM50 subtypes) [8,9]. Results from these studies showed that the gene signatures tend to have better prognosis significance than traditional pathological assessment [7]. This might be due to the integration ability of these gene signatures. For instance, PAM50 can combine the information from the tumor stage, tumor grade and tumor subtype together [9]. However, the known gene signatures are only based on single genomic data source such as gene expression. This might be not adequate since other types of genomic data such as copy number alterations should also include important cancer prognosis information [9]. Advanced algorithms now give us new tools to explore the possibility of integrating different data sources together. For example, Chi, et al. identified several genes and pathways with a high prognostic significance for young breast cancer patients based on their gene expression and copy number alteration data using a graph-based machine learning (ML) method [9].

Traditional ML methods such as artificial neural networks (ANN) and support vector machines (SVM) may suffer some problems in dealing with the high-dimensional, noisy and massive genomic data [10]. Recently, a special case of ANN with more nodes and layers has emerged as an efficient method to handle these high-dimensional and noisy data. The idea of ANN was originated from the information processing and communication patterns in a human nervous system [11]. As the new development of the traditional ANN, deep learning (DL) presents a large group of interconnected artificial neurons with many more layers. Like other learning methods, DL could be implemented in a supervised or unsupervised way, which depends on whether the input data is labeled or not. Although both supervised and unsupervised DL algorithms have been successfully applied to the analysis of genomic data, they could be used to solve different biology problems. Supervised learning algorithms are often used to predict gene functions and gene-gene interactions or to identify new driver genes [12], while unsupervised learning algorithms are often used to cluster the strong signals in the data [13,14]. Among the unsupervised learning algorithms, autoencoder is a new technology that uses the data itself as the learning objective or label. Therefore, it is also known as self-labeled or self-supervised deep learning. Traditional autoencoders may face the invalid learning problem when the number of hidden nodes is larger than the input size. To avoid this potential risk, denoising autoencoders (DAs) came up with the solution of adding some noise into the input data on purpose.

Vincent, et al. brought the concept of DAs into DL and built a specialized feature extraction DL architecture [15]. The key idea of DAs as mentioned above is to add random noise into the raw data before it is input into the network. After the encode and decode processes, the raw data would be reconstructed from the noisy data, while the compact and efficient representations from the raw data could be learned as well [15]. These representations are the DAs-based genomic features.

DL as a special case of ML and ANN has been applied to mine deep information from complex genomic data and has generated interesting results [16]. Its high integration and reconstruction abilities give us large flexibility to combine different types of genomic data to extract valuable information from them. It has been expected that deep features extracted by DL models would perform better in clinical association and prognosis prediction than standard gene or pathway signatures [17]. For example, Tan, et al. reported a deep feature representing ER status and a deep feature with high prognosis significance based on breast cancer gene expression data [13]. These deep features were constructed by a DAs and performed better in the downstream analyses [13]. However, these studies were based on only a single genomic source.

**Figure 1:**
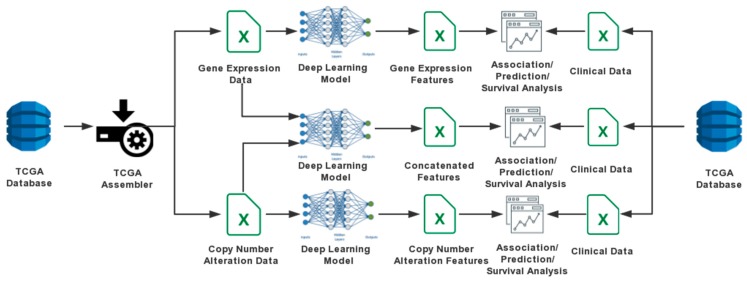
A flowchart illustrating the analysis procedures in this study.

This study aims to extract the integrated features from both the gene expression and CNA data by a concatenated DAs model. As a comparison, we also built a standard DAs model to extract deep features from gene expression and CNA data separately. The comparisons were made in terms of the performance in association analysis as well as prognosis analysis. The study design and analysis procedures are shown in Figure 1.

## 2. Materials and Methods

### 2.1. Data Sources

Datasets used in this study came from The Cancer Genome Atlas (TCGA) [18], which is one of the most comprehensive genomic databases. TCGA provides 1098 breast cancer patients’ clinical data along with their genomic data. These genomic data include gene expressions, CNA, protein expressions, micro RNA (miRNA) expressions, and somatic mutations.

For gene expression data, the sequencing, alignment, quality control and quantification were performed previously [18]. Using the TCGA-Assembler tool [19], we downloaded the gene expression raw count, then filtered out unexpressed genes and those genes with a count per million (CPM) less than 1 in 3 patients. We performed normalization of the data using Upper Quartile Fragments per Kilobase of transcript per Million mapped reads (FPKM-UQ) [20]. FPKM-UQ is a modified FPKM algorithm in which the total read count is replaced by the 75th percentile read count for a given sample. 

Similar to the gene expression data, upstream processes of CNA data were done previously as well [18]. Using the downloaded chromosome-region specific log2 copy number data, we calculated the gene-level CNA values using the TCGA-Assembler tool. Several data cleaning procedures such as removing all-NAs were also performed to avoid potential format issues in the follow-up analysis.

After normalization and preprocessing, there were 18,163 genes from each of the 1095 patients left for gene expression data and 23,563 genes from the 1098 patients left for CNA data. To keep the gene dimension and scale matched in the two data sources, both of them were linearly transformed into a range between 0 and 1, resulting in the decreasing of the data dimension to 16,197 (genes) × 1085 (patients) for both data sources.

### 2.2. DA Models

**Figure 2:**
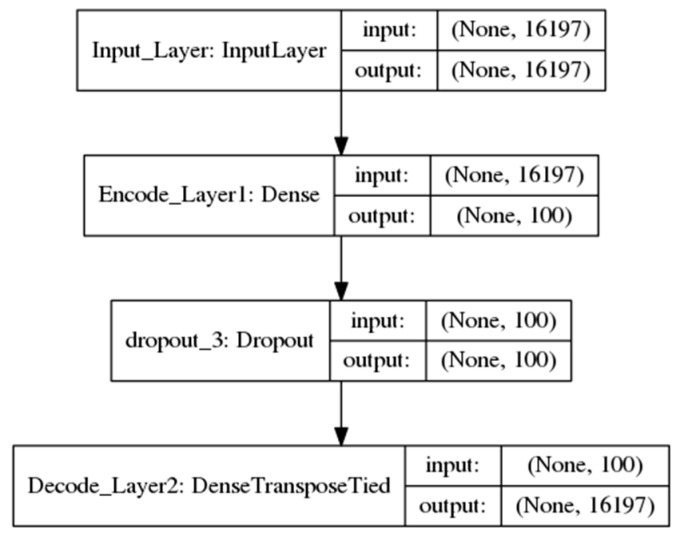
The one-input denoising autoencoders model. There are two hidden layers in the encode phase and two decode layers. The input can be either gene expression data or copy number alteration data.

**Figure 3:**
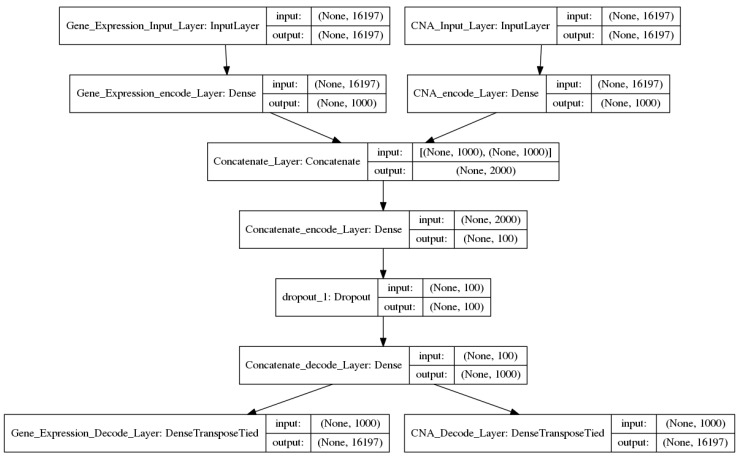
The two-input DAs model. There are two hidden layers in the encode phase and one decode layer. Concatenation was performed between the two encode layers.

Two DAs models were developed using Keras [21] with Tensorflow [22] as the backend to extract deep genomic features. One model was for feature extraction from a single genomic source, named as one-input DAs model (Figure 2). The other, named as the two-input DAs model (Figure 3), was for concatenated feature extraction from the integrated genomic sources.

#### 2.2.1. One-Input DAs Model

This architecture was composed of one input layer, one fully connected encode hidden layer with 100 nodes which were chosen to be the deep features used in this study and one decode layer which uses the transpose of encoding layer’ weights. This procedure can be formulated as below:

encode = sigmoid (*W* × input + *b*) decode = sigmoid (*W’* × encode + *b*’) (1)

where *W* is the weight metrics between the layers with the size of 16,197 × 100, *b* is the bias for each node, and the sigmoid function is sigmoid (*x*) = 1 / (1 + *e^−x^*). The counterparts with the superscript refer to the transpose metrics. A dropout layer was added after the encode layer, which randomly set 50% of the output of encode layer to 0 to prevent overfitting. The encode item was chosen to be the activity values of the deep features in this model.

#### 2.2.2. Two-Input DAs Model

Literally, the two-input DAs model contained two input layers, followed by one encode layer with 1000 nodes for each input layer, then followed by a concatenated layer, and another encode layer with 100 nodes which were chosen to be the deep concatenated features. Finally, there were two decode layers. The procedure can be formulated as follow:

input_1__encode_1_ = sigmoid (*input_1__W_1_* × input_1_ + *input_1__b_1_*) input_2__encode_1_ = sigmoid (*input_2__W_1_* × input_2_ + *input_2__b_1_*) concate_encode_1_ = concatenate (input_1__encode_1_, input_2__encode_1_) concate_encode_2_ = sigmoid (*concate_W_2_* × concate_encode_1_+ *concate_b_2_*) output_1_ = sigmoid (*input_1__W_1_′* × concate_encode_2_ + *input_1__b_1_′*) output_2_ = sigmoid (*input_2__W_1_′* × concate_encode_2_ + *input_2__b_1_′*) (2)

where *input_1_*_*W_1_*, *input_2_*_*W_1_*, and *concate_W_2_* are the weight metrics between the layers with the size of 16,197 × 1000, 16,197 × 1000, 2000 × 100 respectively. The *input_1_*_*b_1_*, *input_2_*_*b_1_*, and *concate_b_2_* are the biases for each node. The counterparts with superscript refer to the transpose metrics. A dropout layer was added after concate_encode_2_ layer, which randomly set 50% of the output of that layer to 0. The concate_encode_2_ was chosen to be the activity values of the deep features in this model.

### 2.3. Train the Models

Before the training process, the input data sets were disrupted by a noise factor of 0.25, which is the proportion of the number of genes in the data sources. These genes were selected randomly and their values were set to 0. The binary cross-entropy function shown below was used to measure the difference between the input layer and the output layer:

*L* (*input*, *output*) = −(1/*N*) Σ (*input_k_* × *log*(*output_k_*) + (1 − *input_k_*) × *log*(1 − *output_k_*)) (3)

where *L (input, output)* is the binary cross-entropy, *K* is the index of batches, *N* is the total number of batches. Thus, the training task is to minimize the *L (input, output)*.

For the optimizer, e.g., the strategy to update the weights and bias so that the minima could be found, we selected stochastic gradient descent (SGD), which has several arguments to be set freely. After having different trials, the learning rate was finally set to 0.1; the batch size and epoch were set to 64 and 100 respectively. The models were finally trained under the parameters mentioned above. The activity values and weight metrics related to deep features were read out.

### 2.4. Visualization and Clustering

Heatmap3 [23] was used to visualize the activity values of these deep feature sets. We used the complete linkage function in the hierarchical clustering process and visual-guided criteria by analysis of the dendrogram to decide the number of clusters. First, the clinical data downloaded from TCGA were carefully scanned and the most clinical-relevant characteristics such as pathological status (T, N, M), tumor stage, estrogen receptor (ER) status, progesterone receptor (PR) status, human epidermal growth factor receptor 2 (HER2) status, triple negative status, and PAM50 subtypes (i.e., Luminal A, Luminal B, Basal-like, HER2-enriched, and Normal-like) were extracted. These clinical characteristics were shown as the sidebar of the heat map.

### 2.5. Association Analysis

To test whether the identified patient clusters are associated with known clinical and molecular characteristics, we applied both Fisher’s exact test and Pearson’ Chi-square test.

Survival differences between the identified patients groups were evaluated by Kaplan–Meier (KM) curves. Furthermore, associations between each deep feature in the three feature sets (gene expression, CNA, the concatenated one) and patient’s overall survival was assessed using Cox’s proportional hazards (COX-PH) model [24]. The hazard function is

*h*(*t*) = *h*_0_ (*t*) × *exp* (*bx*) (4)

where *t* represents the survival time. *b* is the coefficient which measures the impact of the covariate *x.* Later, a risk score for each feature set was generated from the COX-PH coefficients:

*r* = Σ (*b_i_* × *a_i_*) (5)

where *r* is the risk score, *b_i_* is the coefficient from the COX-PH model and *a_i_* is the related activity value of the given feature. Afterward, the risk scores were binarized into high-risk and low-risk groups using R package xtile function with a *prob* parameter set to 0.55, which means we use the 55% quantile as the cutoff to bin the patients into the high-risk and low-risk groups. Finally, the survival differences between these two groups were evaluated by the KM curve.

### 2.6. Gene Sets Enrichment Analysis

For each of the three DAs models for gene expression, CNA and their concatenated one, we traced back their gene-level weights based on

*W_g_* = *W* × *B* (6)

where *W* is the 16,197 × 100 dimensional weights that were extracted from a given DAs model previously. *B* is the vector of COX-PH coefficients. The gene-specific weights *W_g_* were filtered by a cutoff 0.01, which resulted in the three selected gene lists with 6954, 5381 and 6297 genes, respectively. Finally, the three gene lists were used to perform gene set enrichment analysis (GSEA) by the Enricr tool [25] to identify the up-regulated and down-regulated pathways. Kyoto Encyclopedia of Genes and Genomes (KEGG) [26] and Gene Ontology (GO) [27] Biological Process 2018 version were chosen to be the reference gene sets.

## 3. Results

**Figure 4:**
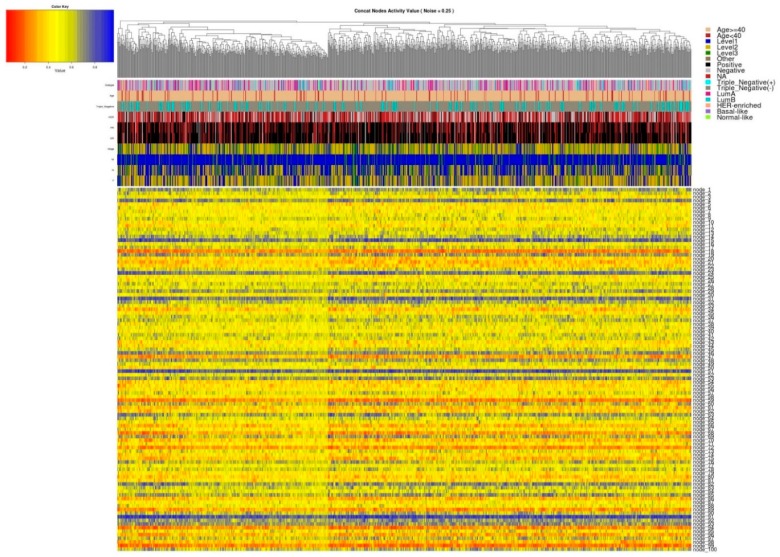
The clustering of activity values of concatenated deep features extracted under the noise factor of 0.25. The columns are the 1085 patients and the rows are the 100 deep features. The sidebar contains the corresponding clinical information of the patients. Values are clustered by both columns and rows.

Based on the normalized and processed breast cancer genomic data, our models were trained and the activity values of the 100 deep features for each of the three data sets as well as the weights matrices were extracted (Table 1). Then we clustered these activity values for each of the three data sets. Overall, there were no clear patterns shown in the deep features from a single genomic source (gene expression or CNA data). However, patients were roughly clustered into 2 groups according to the activity values of the concatenated deep features (Figure 4).

**Figure 5:**
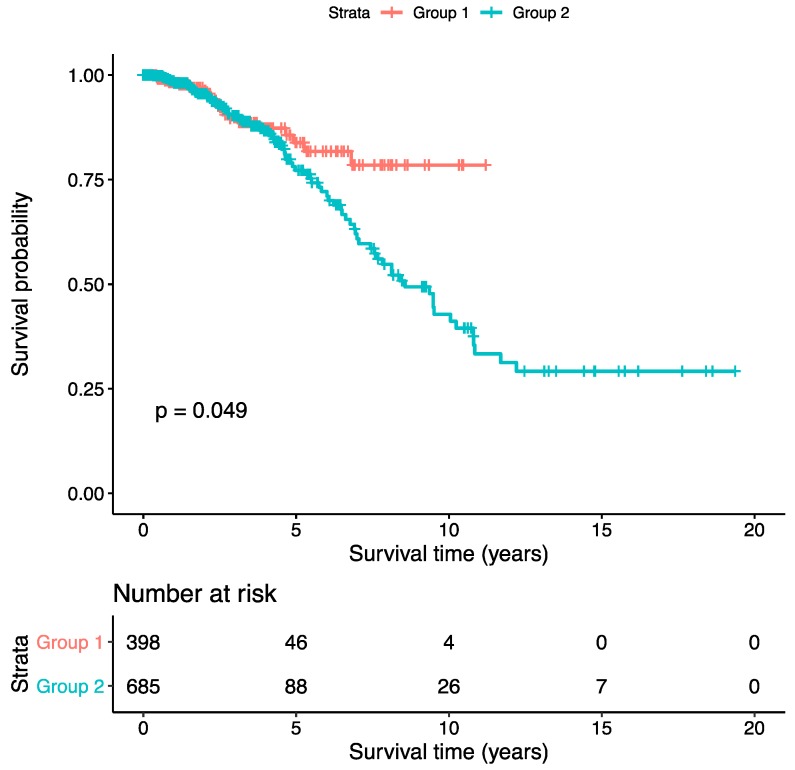
The Kaplan–Meier (KM) plot of the two patient groups clustered by the concatenated features.

Results from the association tests between the two patient groups and their clinical characteristics are shown in Table 2. The two patient groups showed significant survival (Figure 5) and ER status difference (Table 2) with *p*-values 0.049 and 0.002, respectively, which mean that the concatenated features have learned the ER information and performed well in predicting patient’s prognosis. The odds ratio of ER status is 0.626, indicating that the second group tends to be associated with ER-negative patients. From the KM plot (Figure 5), we can see that the patients in Group 2 suffered from a poor prognosis, which happens to be associated with ER-negative status. It has been shown that ER-negative breast cancer patients usually have a poor prognosis.

**Figure 6:**
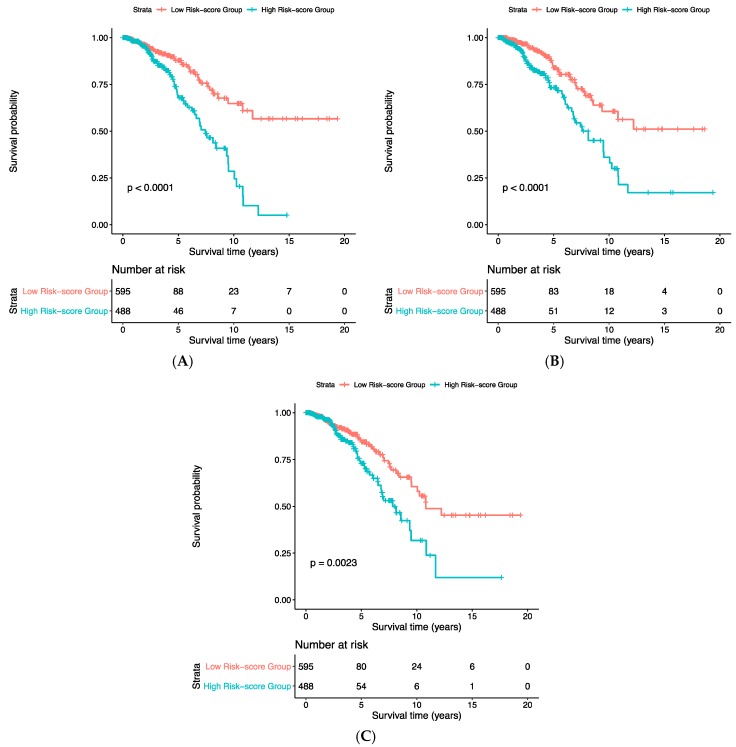
The KM-plots for risk scores based on the deep feature sets. (**A**) Gene expression data; (**B**) copy number alteration data; (**C**) the concatenated data.

According to the results of COX-PH models, the high-risk scores generated from each of the three deep feature sets are all significantly associated with a poor overall survival with *p*-values less than 1 × 10^−5^ (Table 3). The concatenated features showed a higher hazard ratio (HR) with 95% confidence interval (CI) (1.27, 1.16–1.40) than gene expression features (1.009, 1.005–1.013) and CNA features (1.23, 1.15–1.32). These results indicated that the risk scores from the deep features, especially the concatenated risk score, predict patient’s poor prognosis. The similar patterns were observed in KM plots (Figure 6), where the patient group with high-risk scores always suffered from a poor prognosis.

GSEA using the 6,297 genes selected based on the concatenated deep feature set showed that the AMP-activated protein kinase (AMPK) signaling pathway in the KEGG family was significantly down-regulated with a false discovery rate (FDR) less than 0.05, and several GO-based regulation processes, such as the regulation of DNA-templated transcription, the regulation of nucleic acid-templated transcription, the regulation of apoptotic process, the positive regulation of gene expression, the positive regulation of cell proliferation, and the regulation of cellular macromolecule biosynthetic process were significantly enriched as well, with an FDR less than 0.05 (Table 4).

AMPK is an important cellular metabolism and energy homeostasis regulator in mammalian tissues. It is situated in the center of a signaling network which contains tumor suppressors such as LKB1, TSC2 and p53 [25]. Some evidence has been reported that AMPK plays an anti-tumorigenic role and a lot of work are ongoing to involve agonists of AMPK for cancer treatment [28]. Furthermore, all those enriched GO regulation processes are critical as hallmarks in cancer occurrence and progression [29].

## 4. Discussion

In building the DA model, one of the key parameters we need to set up is the noise level used to partially destroy the inputs. We tried to add different levels of noise (e.g., 0%, 10%, 25% and 50%) into the DA model. Similar to the observations made by Vincent et al. [15], we also found that the more noise was added the better the network learns dependencies between the features. With low noise levels, the learned features do not stand out. As we set the noise level at 0.25, denoising training can capture more distinctive deep features.

Comparing with conventional breast cancer biomarkers, such as CA15-3 for measuring how breast cancer treatment is working and looking for cancer that has come back or recurred, after treatment [30], and NCC-ST-439 for measuring breast cancer progression [31], the explanation of the deep genomic features or biomarkers from the DA model for breast cancers is more complicated. Each of the extracted deep features is a high-level summary of the raw features or conventional biomarkers. These high-level features or biomarkers can be more robust against noise in the conventional biomarkers. Furthermore, these high-level features can potentially significantly improve the breast cancer outcome prediction by integrating information from both breast cancer histology images and genomic biomarkers [32]. The extracted features based on the proposed deep learning model can be also used to predict the statuses of malignancy, relapse, and reactivity for anticancer if the related data sources are available. We will explore the method in other large data sets and cancer types in the future. This can further validate the usefulness of the method for risk stratification of cancer patients.

In order to extract robust deep features using the proposed DA model, we took a strategy to add noise into the input genomic data by the partial corruption of the input pattern. It is expected that the learned deep features from the partially destroyed inputs can yield almost the same representation of the raw genomic data. In order to further boost the performance of using the learned deep features to predict breast cancer outcome or traits, another interesting strategy is to incorporate the prior knowledge about breast cancer hallmarks, which can be represented by a few molecular or signaling networks [33], into the deep learning procedure. This can be potentially implemented in different ways. For example, the interaction information among different genes or mutations collected in the molecular or signaling network databases can be used to assign the weights in different layers of the network among different neurons. We will explore the interesting strategy in future studies.

## 5. Conclusions

In this study, we showed that unsupervised DAs as an effective model to extract meaningful deep genomic features from either single- or multi- genomic sources from breast cancer patients. These features were significantly associated with the breast cancer ER status and had the prognosis significance. We also showed that the concatenated deep features were enriched by breast cancer relevant pathways.

This study can be improved in two potential ways. The first one is to develop new DAs model structures such as stacking more layers into DAs or adding a regression layer to make it supervised [34]. The second one is to combine all types of possible data sources together, such as somatic mutation data, protein expressions, miRNA expressions, etc. We will explore these ideas in future analyses.

## Figures and Tables

**Table 1:** The size and organization of deep features obtained from the models. The size and structure of the deep features extracted from gene expression data and copy number alteration data by the two denoising autoencoders (DAs) models.

Model	Data Source	Deep Features (Noise Factors = 0.25)	
One-input DAs	Gene expressions	Activity values	1085 × 100
		weights	16,197 × 100
	Copy number alterations	Activity values	1085 × 100
		weights	16,197 × 100
Two-input DAs	Gene expressions Copy number alterations	Activity values	1085 × 100
		weights	16,197 × 100

**Table 2:** The results of clinical association analysis. * patients were classified as young (age < 40) and old (age ≥ 40) groups.

Clinical Characteristics	Fisher’s Exact *p*-Value	Chi-Square Test *p*-Value
Pathological T	0.69	0.69
Pathological N	0.95	0.96
Pathological M	0.95	0.94
Tumor Stage	0.93	0.93
ER Status	0.002	0.002
PR Status	1.00	0.99
HER Status	0.43	0.44
Age *	0.58	0.67
Triple Negative Status	0.15	0.17
Tumor Subtype	0.35	0.36

**Table 3:** Cox’s proportional hazards (COX-PH) results for risk scores.

Risk Score	HR	Lower.95_HR	Upper.95_HR	*p*-Value
Gene expression	1.009	1.005	1.013	1.06 × 10^−5^
CNA	1.23	1.15	1.32	7.86 × 10^−9^
Concatenated	1.27	1.16	1.40	5.62 × 10^−7^

**Table 4:** The gene set enrichment analysis using Enricr.

Gene Sets	*p*-Value	Adjusted *p*-Value
regulation of transcription, DNA-templated	2.25 × 10^−7^	0.001
regulation of nucleic acid-templated transcription	6.03 × 10^−5^	0.04
regulation of apoptotic process	6.19 × 10^−5^	0.04
positive regulation of gene expression	5.89 × 10^−5^	0.04
positive regulation of cell proliferation	4.78 × 10^−5^	0.04
AMPK signaling pathway_Homo sapiens_hsa04152	6.08 × 10^−5^	0.018

## Abbreviations

DL	Deep learning
CNA	Copy number alteration
DAs	Denoising autoencoders
TCGA	The Cancer Genome Atlas
KM	Kaplan-Meier
COX-PH	Cox’s proportion hazard
OR	Odds ratio
AMPK	AMP-activated protein kinase
FDR	False discovery rate
GSEA	Gene sets enrichment analysis
ML	Machine learning
ANN	Artificial neural network
SVM	Support vector machine
ER	Estrogen receptor
miRNA	Micro RNA
CPM	Count per million
FPKM-UQ	Upper quartile fragments per kilobase of transcript per Million mapped reads
SGD	Stochastic gradient descent
KEGG	Kyoto Encyclopedia of Genes and Genomes
GO	Gene Ontology
